# Magnitude, characteristics, and factors associated with severe occupational injuries among patients presenting to emergency departments in Addis Ababa, Ethiopia

**DOI:** 10.1186/s12873-026-01643-3

**Published:** 2026-06-11

**Authors:** Hailemichael Mulugeta, Bente Elisabeth Moen, Teferi Abegaz, Abdisa Oli, Mengistu Mekonnen, Hana Ketema, Wakgari Deressa

**Affiliations:** 1 Department of Public Health, School of Public Health, College of Health Sciences, Debre Berhan University, Debre Berhan, Ethiopia; 2 Department of Preventive Medicine, School of Public Health, College of Health Sciences, Addis Ababa University, Addis Ababa, Ethiopia; 3 Department of Global Health and Primary Health Care, Centre for International Health, University of Bergen, Bergen, Norway; 4 Emergency Department, Tirunesh Beijing General Hospital, Addis Ababa, Ethiopia; 5 Academic and Research Directorate, ALERT Hospital, Addis Ababa, Ethiopia; 6 Department of Emergency and Critical Care, AaBET Hospital, Addis Ababa, Ethiopia; 7 Department of Epidemiology and Biostatistics, School of Public Health, College of Health Sciences, Addis Ababa University, Addis Ababa, Ethiopia; 8 Aklilu Lemma Institute of Health Research, Addis Ababa University, Addis Ababa, Ethiopia

**Keywords:** Emergency department, Ethiopia, Injury severity, Occupational injury

## Abstract

**Background:**

Occupational injuries account for more than 395 million cases and almost 3 million deaths globally each year, with the highest burden occurring in low- and middle-income countries. In Ethiopia, occupational injuries contribute substantially to emergency department visits, yet evidence on the magnitude, characteristics, and determinants remain limited. This study aimed to describe the magnitude and characteristics of occupational injuries and identify factors associated with severe injuries among patients presenting to emergency departments.

**Methods:**

A prospective hospital-based observational study was conducted at three urban public hospitals in Ethiopia. A total of 868 participants aged 18–60 years who visited emergency departments with occupational injuries were consecutively enrolled. Data was collected from October 3, 2024, to March 14, 2025, using structured interviews and clinical chart reviews. Injury severity was classified based on the World Health Organization injury surveillance guideline. Descriptive statistics summarized patient characteristics, work, and injury patterns. Poisson regression with robust variance estimation was used to identify factors associated with severe injury, and adjusted prevalence ratios with 95% confidence intervals were reported.

**Results:**

Among 5,038 injury patients, 918 (18.2%) had occupational injuries. Of these, 868 (94.6%) participated in the study. The median age was 27 years, and most participants were male (89.5%). The majority were employed in manufacturing (43.9%) and construction (24.0%). Craft and related trades (34.9%) and elementary workers (34.2%) were the most affected. Upper extremity injuries were the most common (57.0%), with lacerations (48.2%) as the leading nature of injury, followed by fractures (28.7%). The primary mechanism of injury was contact with sharp, pointed, or rough elements (43.9%), followed by falls or crashes into objects (16.6%). Among the patients, 175 (20.2%) had severe injuries. Low educational level, fall-related mechanisms, no fixed work area, and accidents due to peers and organizational causes were significantly associated with severe injury in a multivariable regression analysis.

**Conclusions and recommendations:**

Occupational injuries are a burden on emergency departments, affecting a high proportion of young, male workers in the construction and manufacturing sectors. Severe injuries were related to education, type of work, and injury mechanism. This study underscores the need for targeted workplace safety interventions.

**Supplementary Information:**

The online version contains supplementary material available at 10.1186/s12873-026-01643-3.

## Background

Occupational injuries are a large global problem, and contribute to 2.93 million worker deaths annually and 395 million non-fatal injuries worldwide in 2019 [1]. An occupational injury is defined as any personal injury, illness, or death resulting from an occupational accident [2]. While global analyses indicate a decline in the occupational injury-related mortality rate between 1990 and 2019 [3], and the distribution of these injuries varies widely among regions. Notably, low- and middle-income countries (LMICs) accounted for 92% of global unintentional occupational injury deaths in 2016 [4].

These disparities become even more apparent when examining regional patterns. According to the World Health Organization (WHO) and the International Labour Organization (ILO), the burden of occupational injuries in 2021 was highest in the African, South-East Asia, and Western Pacific regions compared with Europe and the Americas [5]. In sub-Saharan Africa, occupational injury-related mortality has continued to rise despite global improvements [3]. Non-fatal injuries are also common, with a pooled prevalence of 57.0% among workers in high-risk sectors such as construction, manufacturing, and mining [6]. Temporary employees and workers without safety training are particularly vulnerable [7]. These trends underscore the need for robust, region-specific data to guide prevention and emergency strategies.

Ethiopia, a low-income country, reflects these regional patterns and faces a similarly high burden of occupational injuries. A meta-analysis of studies conducted between 2009 and 2018 estimated a pooled prevalence of 39.3% among workers in the manufacturing, construction, and waste management sectors [8]. More recent studies (2019–2023) in construction [9, 10], manufacturing [11–14], mining [15], and other service sectors [16–19] reported a mean prevalence of 41.3%, ranging from 10.7% in textile industries [11] to more than 64.0% in construction [9] and small-scale industries [14]. Factors associated with occupational injury in Ethiopia include sociodemographic characteristics (male sex, young age, low educational level, limited work experience) [9–11, 16, 20]; behavioral factors (khat chewing, smoking, sleep disturbances) [11, 13, 16, 18]; and work-related factors such as lack of safety training, inadequate availability of personal protective equipment (PPE), excessive working hours, hazardous job categories, absence of supervision, insufficient workspace, temporary employment, low safety climate, workplace stress, and poor job satisfaction [11–13, 15–17, 20–22].

Despite these important contributions, existing Ethiopian studies share a key limitation; they are all workplace-based surveys relying on self-reported data from healthy workers and lack clinical verification. This limitation is particularly consequential for severe injuries, here defined as those requiring intensive medical or surgical care in a hospital, and is more likely to result in long-term disability or death [23]. Severely injured workers are less likely to participate in workplace surveys because they may be recovering at home or may have died before data collection [24]. Consequently, workplace-based studies likely underestimate the true burden of occupational injuries and the factors that influence injury severity. Hospital-based studies, by contrast, can overcome these limitations by providing clinically confirmed diagnoses and capturing both severe and non-severe injuries, and identifying determinants of severe injury [25–29].

Evidence from other countries underscores the importance of hospital-based data, particularly from emergency departments (EDs), which serve as critical entry points for acute injury care. Studies have reported that occupational injuries account for 4.4% of ED visits in India [30], 11.9% in Canada [28], and 30.7% in Qatar [25].

In sub-Saharan Africa, EDs manage a high volume of injury cases [31, 32]. However, despite this substantial case load, the magnitude and characteristics, and associated factors with severe occupational injuries presenting to EDs are rarely documented. This gap is especially pronounced in Ethiopia, where no recent hospital-based studies have systematically examined occupational injuries in EDs. The absence of such data limits policymakers’ and clinicians’ ability to design targeted interventions, allocate emergency care resources effectively, and prioritize strategies to reduce the burden of occupational injuries.

Therefore, this study aimed to determine the magnitude and characteristics of occupational injuries among patients presenting to the emergency departments of three hospitals in Ethiopia. In addition, the study assessed factors associated with severe injuries in this population. The findings will provide essential evidence to inform policymakers and healthcare providers and offer baseline data to support the development of interventions to improve occupational health and safety in low-income settings.

## Methods and materials

### Study design and setting

A prospective, hospital-based observational study was conducted from October 3, 2024, to March 14, 2025, at three government-owned urban hospitals in Addis Ababa, the capital city of Ethiopia. The study hospitals included Addis Ababa Burn, Emergency, and Trauma (AaBET), All Africa Leprosy Rehabilitation and Training Center (ALERT), and Tirunesh Beijing General Hospital (TBGH). AaBET and ALERT are tertiary-level, major trauma referral hubs in Ethiopia, providing specialized emergency, surgical, and critical care for injury patients presenting directly or referred from other health facilities. TBGH hospital is a secondary-level general hospital; it provides injury care and ranks third in trauma patient volume after AaBET and ALERT hospitals, and receives most patients from Addis Ababa and the adjacent areas.

### Study population, participants and recruitment procedure

Study population: All patients aged 18 to 60 years who presented at EDs with occupational injuries during the study period (from October 3, 2024, to March 14, 2025) were considered for participation in the study.

Inclusion criteria: Data collectors screened the eligible patients in the EDs using the ILO occupational injury criteria [2] after the initial clinical assessment was done by the ED nurses. All patients with occupational injuries were included. The patient was considered to have an occupational injury if the injury occurred while performing work-related tasks or duties, regardless of whether they sustained one or multiple injuries.

Exclusion criteria: Patients with injuries sustained while commuting to or from work were excluded because such events fall outside working hours and are not considered occupational [2]. Patients who returned to the ED for follow-up visits related to the same injury were only registered once.

### Data collection tools and procedures

After deciding that a patient had experienced an occupational injury, data were collected using a structured interview questionnaire and a data extraction checklist. A structured interview questionnaire was used to collect information on sociodemographic characteristics, occupation, sectors, workplace, and accident-related factors. The tool was developed from a previous protocol for a hospital-based prospective observational study [34] and ILO Household Occupational Injury Survey Guidelines (pp. 105, 134–135) [2], with modifications to fit the hospital-based context. A data extraction checklist was used to collect clinical data, including injury characteristics, treatments, surgery, Intensive Care Unit (ICU) admission, and discharge outcomes. It was adapted from the WHO Injury Surveillance Guidelines (pp. 20, 35, 76) [23] and the ILO guidelines (pp. 105) [2].

The data collection team consisted of 22 members. Seventeen BSc nurses conducted patient interviews and extracted data from medical charts in the EDs. Two data clerks recorded the total number of injury cases from the trauma registration logbooks. Three senior clinical doctors supervised the overall process. The nurses and supervisors were formally assigned to the study hospitals by the ED heads and the respective hospital research and ethics offices. They received training on the study procedures and research ethics before data collection began. All data collection activities were conducted independently of their routine clinical duties to avoid any conflict with patient care. Data collection began at ALERT Hospital on October 3, 2024, followed by AaBET Hospital on October 22, 2024, and TBGH Hospital on October 30, 2024, and was conducted simultaneously at all sites until March 14, 2025. This approach allowed the team to familiarize themselves with the procedure and to apply a uniform data collection approach in study hospitals.

Data collection was conducted 24 h a day, 7 days a week in all hospitals. All injury cases presenting to the EDs were recorded in the trauma registration logbooks. Patients sustaining occupational injury [2] were interviewed.

The interviews were conducted once patients were clinically stable and had received necessary medical care. Interviews were administered face-to-face in either Amharic or Afaan Oromo, based on the language of patient spoke (S1, S2, S3). These two languages are the main local languages in Ethiopia. For patients unable to respond, proxies (who are responsible for the patients’ care, either in the hospital or at home) were interviewed instead.

If neither the patient nor proxy could be interviewed in the hospital due to severe injury, discomfort, or loss to follow-up, data collectors conducted phone interviews within one week. In the case of in-hospital or on-arrival deaths, a proxy interview was conducted 15 days after the burial.

Clinical data were routinely documented by attending physicians or ED clinical management teams. AaBET and ALERT hospitals used paper-based medical records, while TBGH used an integrated electronic medical record system. Accordingly, clinical information was extracted from paper charts at AaBET and ALERT hospitals and from electronic databases at TBGH hospital. For each patient, the physicians documented the number of injured anatomical or body parts, the specific anatomical site(s) affected, the injury outcome, and the treatment provided.

### Study variables

The primary outcome was severe occupational injury (Yes/No). Injury severity was classified following WHO guidelines [23] and a prior study [31]. Injury severity was classified at the time of hospital discharge, as follows by the data collectors based on the patient’s clinical data:

- **Minor**: Injuries with no visible signs of injury on the body; superficial injuries such as bruises or superficial lacerations.
- **Moderate**: Injuries extending beyond subcutaneous tissue but not involving visceral organs, requiring some skilled medical treatment.
- **Severe**: Injuries requiring intensive medical or surgical management (e.g., internal hemorrhage, punctured organs, severed blood vessels injury) or resulting in death.

The independent variables were categorized into four domains:

- **Sociodemographic variables**: sex, age in years, education level (four categories), marital status (married and separated/divorced/widowed), and place of residence (by region where they currently live).
- **Occupational/organizational variables**: sector (the economic activity in which a person works) was categorized into eight according to the *International Standard Industrial Classification of All Economic Activities (ISIC)* [2].The ownerships of the sectors were private/corporate, self/family, and public or government; and the work experience was in years. Participants’ occupations were meticulously categorized into five categories using the *International Standard Classification of Occupations (ISCO-08)* [2].
- **Accident-related variables**: patients were asked about the place of the accident, categorized as follows: in the establishment, including farms or holdings for agricultural workers; or in a work area away from the establishment, including a location with no fixed work area (e.g., offsite work, street vendors, shoe-shiners, drivers, etc.) [2]. The contributing cause of injury was assessed through a self-reported question: *‘What do you believe was the primary cause of the accident?’* The response with options included: lack of training, lack of work experience, worker fatigue, personal negligence, errors by other workers, poor equipment maintenance, and an option for ‘other’ [35]. For analysis, causes were categorized into four: worker-related, co-worker/peer-related, and organizational/system-related. Worker-related causes included reasons related to the patient, such as fatigue, negligence, or lack of work experience. Co-worker or peer-related causes refer to injuries resulting from another person’s distraction or error in judgment. Organizational or system-related causes referred to systemic or technical/mechanical factors in the workplace, including lack of PPE or any safety materials, inadequate training, safety failures, work speed pressures, faulty or poorly maintained equipment, and unsafe working areas. Furthermore, days, times, and uses of PPE or safety materials for injury prevention at the time of the accident were collected.
- **Injury-related variables**: injured anatomical parts, nature of injury, injury mechanism, surgery intervention, ICU admission, length of hospital stay in days and hours, and discharge status. If a patient presented with multiple injuries, the most severe was considered for injury characterization.

In this study, the magnitude of occupational injuries was defined as the proportion of occupational injury cases among all injury patients presenting to the EDs, while the magnitude of severe occupational injury was defined as the proportion of severe occupational injury cases among all occupational injuries. Injury characteristics referred to injury-related features of the injured workers.

### Data quality control

The questionnaire was first developed in English and translated into Amharic and Afaan Oromo by health professionals familiar with medical and occupational terminology. Both translated questionnaires were then back-translated into English by other experts to ensure consistency. Only minor discrepancies were identified and corrected for the final questionnaires.

A detailed data collection manual was prepared and distributed to standardize procedures and minimize information bias during data collection. All data collectors participated in a full-day training covering study objectives, tools, interviewing techniques, clinical data collection, and ethical considerations. A pretest of the interview questionnaire and checklist was conducted in one week in 2024 among 45 occupationally injured patients at one hospital before the actual data collection. Following the pretest, a half-day debriefing session was held to discuss challenges and revise the tools.

During the study period, the principal investigator and supervisors closely monitored the procedure, checked the completeness of the data, and provided on-site support. When gaps or inconsistencies were identified, the data collectors conducted phone interviews with patients (15–20 min) or reviewed hospital records to ensure data accuracy.

### Statistical analyses

The data were entered using Epi-Info version 7.1 and then imported into Stata version 17 for statistical analysis. Descriptive statistics (frequencies, percentages, means, medians, standard deviation, and ranges) were used to summarize participant characteristics. The magnitudes of occupational injury among ED injury cases and the prevalence of severe injury were reported with 95% confidence intervals (CIs).

Pearson’s χ² test was used to examine differences in injury mechanisms and severity across hospitals and to assess the relationships of work and accident characteristics with injury severity. Because the prevalence of the main outcome (severe injury) exceeded 10%, Poisson regression with robust variance was used instead of logistic regression, providing the prevalence ratios (PRs) for both bivariable and multivariable analyses [36).

The candidate variables for the multivariable model were selected based on the criteria of *p* < 0.20 in the bivariable analysis and along with theoretically important. Adjusted prevalence ratios (APRs) with 95% CI were calculated. After checking the multicollinearity and model fitness, ten variables were retained in the final model. Multicollinearity was not detected (mean VIF = 1.66; range 1.04–3.54) and model diagnostics also indicated a good fit, as shown by the Deviance (428.5; df = 723; *p* = 1.00) and Pearson chi-square tests (582.41; df = 723; *p* = 1.00).

## Results

During the study period, a total of 5,038 injured patients presented to the EDs. Of these, 4,120 (81.8%) had non-occupational injuries, while 918 had occupational injuries, yielding an occupational injury magnitude of 18.2% (95% CI: 17.2–19.3). Among the 918 eligible patients, 868 (98.6%) completed the interview. Of the respondents, 789 patients were interviewed directly through face-to-face at hospitals, and 41 were interviewed with assistance from caregivers during face-to-face interviews, 18 patients were interviewed by phone after hospital discharge, and 20 proxies were interviewed on behalf of deceased patients. Of the total participants, 377 (43.4%) were from ALERT hospital, 332 (38.3%) from TBGH, and 159 (18.3%) from AaBET hospital.

### Demographic characteristics of the study participants

Most participants in the study were male (*n* = 777, 89.5%), with a median age of 27 years (IQR: 23–35), and the mean age was 29.4 years (SD ± 8.7) (Table 1). Overall, 8.3% had no formal education, and 37.9% had a primary education level (grades 1–8). Most participants (*n* = 692, 79.7%) were from Addis Ababa, followed by Oromia (*n* = 163, 18.8%), and 13 participants (1.2%) from other regions in Ethiopia.

**Table 1:** Demographic characteristics of occupational injury patients (*n* = 868)

Variables	Frequency	Percent
**Sex**		
Male	777	89.5
Female	91	10.5
**Age category**		
18–24	294	33.9
25–29	230	26.5
>29	344	39.6
**Education level**		
No formal	72	8.3
Grades 1–8	329	37.9
Grades 9–12	339	39.1
College/University and above	128	14.7
**Marital status**		
Single/Separated/Divorced/widowed	458	52.8
Married	410	47.2

### Injury characteristics

Most occupational injuries were at a single body part (85.6%), with the upper extremities being the most commonly affected (57.0%) (Table 2) and lacerations were the predominant nature of injury (48.2%). The majority of patients (78.2%) were admitted for less than one day, with a median hospital stay of 5.77 h (IQR: 2.37–20.88), while the mean of stay was 48.0 h (SD ± 172.6). However, 26 (3.0%) remained hospitalized for more than 15 days. A total of 175 patients (20.2%; 95% CI: 17.7–22.9%) were diagnosed with severe injuries. The overall mortality rate was 2.3% (95% CI: 1.3–3.2%). Most patients (64.2%) were discharged home with a follow-up appointment.

**Table 2:** Injury characteristics and clinical outcome (*n* = 868)

Variables	Frequency	Percent
**Body part injured**		
1	743	85.6
2	102	11.8
≥ 3	23	2.6
**Anatomical body site for the injury**		
Head and face	153	17.6
Upper extremities	495	57.0
Lower extremities	125	14.4
Trunk and whole body	95	10.9
**Nature of injury**		
Laceration	418	48.2
Fracture	249	28.7
No visible injury, dislocations, sprains, strains	82	9.4
Amputation	46	5.3
Bum, corrosion, scald	41	4.7
Concussion, internal injury	32	3.7
**Surgery intervention**		
Yes	137	15.8
No	731	84.2
**ICU stay**		
Yes	8	0.9
No	860	99.1
**Length of hospital stay in days**		
< 1	679	78.2
1.00–3.00	92	10.6
3.01-7.00	37	4.3
> 7	60	6.9
**Severe injury status**		
Yes	175	20.2
No	693	79.8
**Discharge status**		
Treated and discharged to home with an appointment	557	64.2
Treated and discharged to home without appointment	236	27.2
Referred to another hospital	31	3.6
Left against treatment	24	2.8
Dead	20	2.3

The most common mechanism of injury was contact with sharp, pointed, or rough elements (43.9%), followed by falls or crashes into objects (16.6%) and being struck by objects (16.5%). There were significant differences across hospitals in injury mechanism, severity, and hospitalization status (all *p* < 0.001). ALERT Hospital received more sharp-object injuries, fall-related injuries, and collision mechanisms of injury than the other hospitals. It also recorded a higher proportion of severe cases and the highest hospitalization rate. Following ALERT, AaBET Hospital also managed a high proportion of severe injuries. Whereas TBGH hospital predominantly treated minor injuries and recorded the lowest hospitalization rate (Table 3).

**Table 3:** Injury mechanisms, severity, and hospitalization across hospitals (*n* = 868)

Variables	Total, *n* (%)	Hospital, *n* (%)			Chi-square
		ALERT	AaBET	TBGH	
**Mechanisms of injuries**					
Contact with sharp/pointed/rough element	381 (43.9)	196 (51.4)	80 (21.0)	105 (27.6)	(χ²) = 74.2 df = 10 *p* < 0.001
Fell or crashed into something	144 (16.6)	64 (44.4)	23 (16.0)	57 (39.6)	
Struck by something	143 (16.5)	38 (26.6)	13 (9.1)	92 (64.3)	
Trapped and stuck by something	73 (8.4)	20 (27.4)	18 (24.7)	35 (47.9)	
Collided with something	55 (6.3)	28 (50.9)	6 (10.9)	21 (38.2)	
Other	72 (8.3)	31 (43.1)	19 (26.4)	22 (30.6)	
**Severity status**					
Minor or superficial	302 (34.8)	87 (29.8)	29 (6.6)	186 (61.6)	(χ²) = 126.7 df = 6 *p* < 0.001
Moderate	391 (45.0)	211 (54.0)	72 (18.4)	108 (37.6)	
Severe but non-fatal	155 (17.9)	69 (44.5)	53 (34.2)	33 (21.3)	
Fatal	20 (2.3)	10 (50.0)	5 (25.0)	5 (25.0)	
**Hospitalization**					
Yes	189 (21.8)	90 (47.6)	65 (34.4)	34 (18.0)	(χ²) = 61.0 df = 2 *p* < 0.001
No	679 (78.2)	287 (42.3)	94 (13.8)	298 (43.9)	

*χ² = Pearson’s chi-square statistic; df = degrees of freedom ﻿

### Injury distribution and severity rate by work characteristics

Of all injuries, the majority of participants, 735 (84.7%) were employed in the private or corporate-owned sector with a median work experience of 3 years (IQR: 1–7 years (Table 4). The leading participants, 381 (43.9), were manufacturing workers, including woodwork, metalwork, textile/garment, food processing, plastics, building materials, and others. The second-largest group of participants, 208 (24.0%), were employed in the construction sector, including building, road, and other construction activities. High proportions of severe injuries occurred among workers in agriculture (40.0%), construction (27.9%) and transport (26.0%). However, the number of from agriculture sector was relatively small (*n* = 15, 1.7%). Regarding the patients’ occupation, craft and trade-related occupations accounted for the largest proportion of participants (*n* = 303, 34.9%), followed closely by elementary occupations (*n* = 297, 34.2%). However, the highest proportion (25.9%) of severe injuries was observed among workers in elementary occupations compared to other occupational groups.

Statistical analysis confirmed that the proportions of severe injuries were significantly different among the sectors (χ² = 20.7, df = 7, *p* = 0.004) and occupations (χ² = 12.6, df = 4, *p* = 0.014).

**Table 4:** Distribution of injuries and severe injury rate by sector, ownership, and occupation (*n* = 868)

Variables	Total, *n *(%)	Severe Injury *n* (%)	Not severe injury *n* (%)	Pearson Chi-Square
**Sectors**				
Manufacturing	381 (43.9)	55 (14.4)	326 (85.6)	χ² = 20.7 df = 7 *p* = 0.004
Construction	208 (24.0)	58 (27.9)	150 (72.1)	
Wholesale, retail trade, restaurants, and hotels	102 (11.8)	19 (18.6)	83 (81.4)	
Private household	63 (7.3)	14 (22.2)	49 (77.8)	
Transport, storage, and communication	50 (5.8)	13 (26.0)	37 (74.0)	
Administrative and support service activities	37 (4.3)	7 (18.9)	30 (81.1)	
Agriculture	15 (1.7)	6 (40.0)	9 (60.0)	
Others	12 (1.4)	3 (25.0)	9 (75.0)	
**Ownership of the sectors**				
Private/corporate	735 (84.7)	149 (20.3)	586 (79.7)	χ² = 0.17 df = 2 *p* = 0.919
Self/family	88 (10.1)	18 (20.5)	70 (79.5)	
Public	45 (5.2)	8 (17.8)	37 (82.2)	
**Occupations**				
Craft and related trades	303 (34.9)	46 (15.2)	257 (84.8)	χ² = 12.6 df = 4 *p* = 0.014
Elementary occupation*	297 (34.2)	77 (25.9)	220 (74.1)	
Plant, drivers, machine operators, and assemblers	192 (22.1)	39 (20.3)	153 (79.7)	
Managers, professionals, technical	41 (4.7)	5 (12.2)	36 (87.8)	
Service and sales	35 (4.0)	8 (22.9)	27 (77.1)	

*ISCO-08 Major Group 9: domestic helpers/housemaids, cleaners, manufacturing laborers, construction laborers, agriculture workers, kitchen assistants, street vendors, refuse collectors, vehicle driver assistance and other helpers/assistants in different sectors

### Injury distribution and severity rate by accident circumstances

Of all injured participants, more than half of the occupational injuries (*n* = 505, 58.2%) occurred within the establishment where the workers were employed (Table 5). However, among workers who were injured at a place away from the establishment or outside any fixed work area had a higher proportion of severe injuries (27.8%) compared with those in establishments (*p* < 0.001). More than half of the participants (58.8%) reported their injuries to be caused by conditions related to the worker, such as fatigue, negligence, or lack of experience. In contrast, a high proportion of severe injuries were reported to be attributed to co-worker factors (27.4%) and organizational/system factors (25.5%) (*p* = 0.001). Furthermore, patients who did not use safety materials (PPE) had a higher proportion of severe injuries (21.5%) compared to safety materials users (*p* = 0.014).

**Table 5:** Distribution of injuries and severe injury rate by place, time, contributing causes, and safety material utilization

Variables	Total *n* (%)	Severe Injury *n* (%)	Not severe injury *n* (%)	Pearson Chi-Square
**Place of accident** (*n* = 868)				
In the establishment	505 (58.2)	74 (14.7)	431 (85.3)	χ² = 22.8 df = 1 *p* < 0.001
Work area away from the establishment or no fixed work area	363 (41.8)	101 (27.8)	262 (72.2)	
**Accident time** (*n* = 868)				
Day (06:01–18:00)	740 (85.3)	144 (19.5)	596 (80.5)	χ² = 5.71 df = 1 *p* = 0.132
Night (18:01–06:00)	128 (14.7)	31 (24.2)	97 (75.8)	
**Accident days** (*n* = 868)				
Week	686 (79.0)	141 (20.6)	545 (79.4)	χ² = 0.31. df = 1 *p* = 0.328
Weekend	182 (21.0)	34 (18.7)	148 (81.3)	
**Contributing cause of accident** (*n* = 743*)				
Workers’ related factors	437 (58.8)	68 (15.6)	369 (84.4)	χ² = 13.5 df = 2 *p* = 0.001
Co-worker /peer factors	157 (21.1)	43 (27.4)	114 (72.6)	
Organizational / system factors	149 (20.1)	38 (25.5)	111 (74.5)	
**Utilization of PPE / safety materials during an accident** (*n* = 866**)				
Yes	173 (20.0)	24 (13.9)	149 (86.1)	χ² = 12.9 df = 1 *p* = 0.014
No	693 (80.0)	149 (21.5)	544 (78.5)	

*Contributing cause of the accident was missing for 125 patients

** Two patients' information about the safety material used during the accident was missing

### Factors associated with severe occupational injury

In the multivariable Poisson regression analysis, educational level, contributing factors to injury, and injury mechanism were significantly associated with severe injury (*n* = 175). Workers with no formal education had more than two times higher prevalence of severe injury (APR = 2.40; 95% CI: 1.55–3.71), and those with primary-level education (grades 1–8) had a 71% higher prevalence (APR = 1.71; 95% CI: 1.22–2.40), compared with those who completed grade 9 or above (Table 6). Severe injury was 40% higher prevalence among workers injured away from the establishment or with no fixed work area (APR = 1.40; 95% CI: 1.01–1.95) than among injuries in the establishment. Similarly, among workers whose injuries were attributed to co-worker or peer-related (APR = 1.62; 95% CI: 1.14–2.29) and organizational or system-related (APR = 1.60; 95% CI: 1.13–2.27) causes had a higher prevalence of severe injury than those who reported worker-related causes. Regarding the mechanism of injury, workers who fell or crashed into something had a 77% higher prevalence of severe injuries compared with among through contact with sharp, pointed, or rough objects (APR = 1.77; 95% CI: 1.18–2.65).

**Table 6:** Multivariable analysis of factors associated with severe injury among occupational injury patients (*n* = 743)

Characteristics	Severe injury *n* (%)		CPR (95% CI)	APR (95% CI)
	No	Yes		
**Sex**				
Male	619 (79.7)	158 (20.3)	1.08 (0.69–1.71)	1.04 (0.66–1.64)
Female	74 (81.3)	17 (18.7)	1	1
**Age category**				
18–24	239 (81.3)	55 (18.7)	0.81 (0.60–1.11)	1.04 (0.69–1.58)
25–29	189 (82.2)	41 (17.8)	0.78 (0.55–1.09)	0.92 (0.63–1.33)
> 29	265 (77.0)	79 (23.0)	1	1
**Education level**				
No formal	46 (63.9)	26 (36.1)	2.68 (1.82–3.93) **	2.40 (1.55–3.71) **
Grades 1–8	243 (73.9)	86 (26.1)	1.94 (1.45–2.60) **	1.71 (1.22–2.40) *
Grades 9 and above	404 (86.5)	63 (13.5)	1	1
**Marital status**				
Single/separate/Widow	376 (92.1)	82 (17.9)	0.79 (0.61–1.03)	0.98 (0.71–1.36)
Married	317 (77.1)	93 (22.7)	1	1
**Occupations**				
Managers, professionals, technical, service, and sales	63 (82.9)	13 (17.1)	1	1
Craft and related trades	257 (84.8)	46 (15.2)	0.89 (0.51–1.56)	1.03 (0.53–2.02)
Elementary occupation*	220 (74.1)	77 (25.9)	1.52 (0.89–2.58)	1.23 (0.66–2.32)
Plant, drivers, machine operators, and assemblers	153 (79.7)	39 (20.3)	1.19 (0.67–2.10)	1.61 (0.84–3.12)
**Work experience in years**				
< 1	143 (84.1)	27 (15.9)	0.71 (0.48–1.06)	0.83 (0.53–1.31)
2–3	256 (80.0)	64 (20.0)	0.90 (0.67–1.20)	0.97 (0.70–1.36)
> 3	294 (77.8)	84 (22.2)	1	1
**Place of accident**				
In the establishment	431 (85.3)	74 (14.7)	1	1
Away from the establishment or no fixed work area	262 (72.2)	101 (27.8)	1.90 (1.45–2.48) **	1.40 (1.01–1.95)*
**Accident time**				
Day (6:01 AM – 12:00 PM)	596 (80.5)	144 (19.5)	1	1
Night (12:01 PM – 6:00 AM)	97 (75.8)	31 (24.2)	1.24 (0.89–1.75)	1.22 (0.87–1.73)
**Contributing factors to injury**				
Workers’ related factors	369 (84.4)	68 (15.6)	1	1
Co-worker/peer factors	114 (72.6)	43 (27.4)	1.76 (1.26–2.46) *	1.62 (1.14–2.29) *
Organizational / system factors	111 (74.5)	38 (25.5)	1.64 (1.15–2.33) *	1.60 (1.13–2.27) *
**Mechanism of injury**				
Contact with sharp/pointed/rough element	323 (84.8)	58 (15.2)	1	1
Fell or crashed into something	94 (65.3)	50 (34.7)	2.28 (1.65–3.16) **	1.77 (1.18–2.65) *
Struck by something	124 (86.7)	19 (13.3)	0.87 (0.54–1.41)	0.99 (0.60–1.63)
Collided with something	39 (70.9)	16 (29.1)	1.91 (1.19–3.08)	1.14 (0.65–2.01)
Others	113 (77.9)	32 (22.1)	1.45 (0.98–2.13)	1.12 (0.73–1.72)

Significant association at ∗*P* ≤ 0.05, ∗∗*P* ≤ 0.01, CI=confidence interval; CPR=crude prevalence ratio; APR=adjusted prevalence ratio

## Discussion

This hospital-based study found that among patients presenting to the emergency departments (EDs) of three public hospitals, occupational injuries accounted for 18.2% of all injury-related presentations; among these 20.2% were classified as severe, and 2.3% resulted in death. Most cases involved young men.

Over two-thirds of the injuries occurred in industrial sectors, with more than half taking place within workplace premises. Workers in craft and related trades were disproportionately affected, and the upper extremities were the most frequently injured anatomical region. Educational level, accident location, contributing causes, and injury mechanisms were significantly associated with injury severity.

The proportion of occupational injuries observed in this study was higher than reported in India (4.4%) [30], but lower than in Qatar (30.7%) [25]. These differences may reflect variations in study settings, case definitions, and data collection methods. The Indian study, conducted in a private teaching hospital, likely underrepresented low-income workers who typically seek care in public facilities. Conversely, the higher estimate from Qatar may be explained by the inclusion of commuting injuries, which likely inflated the reported burden. Moreover, both studies relied on retrospective reviews of medical records, which are prone to incomplete documentation and misclassification.

The current findings highlight occupational trauma as a substantial public health concern and a significant clinical burden within hospital settings. Approximately one in five occupational injuries were severe, requiring advanced surgical or medical management or resulting in death. This underscores the value of hospital-based surveillance in capturing the full spectrum of injury severity, compared with workplace-based studies in Ethiopia that primarily document minor or moderate injuries [9–11, 16, 20]. The observed mortality rate of 2.3% is comparable to reports from Turkey (2.0% and 2.6%) [29, 37], but lower than Qatar (4.3%) [25] and India (3.7%) [33], and higher than Saudi Arabia (no deaths) [26], other studies in Turkey (0.3% and 0.5%) [38, 39], and Bahrain (one death) [40]. Higher mortality in Qatar and India may reflect inclusion of more severe cases or limited pre-hospital and emergency care, whereas very low mortality reports likely stem from methodological and contextual factors rather than a true absence of fatal injuries. These findings highlight the need for strengthened workplace safety systems, improved coordination with emergency and trauma care services, and robust occupational health surveillance.

Recent workplace-based studies in Ethiopia have reported high annual prevalence of occupational injuries in manufacturing [11–14] and construction [9, 10, 20] sectors, with some estimates exceeding 64% [9, 14]. Consistent with these findings, more than two-thirds of occupational injury patients in the current study were employed in these sectors. Similar patterns have been documented in hospital-based studies from Turkey [37], Bahrain [40], and India [33]. These sectors are characterized by limited safety training, weak safety culture, low safety participation, and poor compliance with safety measures factors associated with occupational injuries in Ethiopia [10–12, 20] and likely shared across similar settings. Severe injuries were more common among construction workers, consistent with findings from Jordan [41], where fatal injuries were more frequent in this sector. This reflects the hazardous nature of construction work, where falls and struck-by-object injuries are prevalent [42].

Young men were disproportionately represented among occupational injury patients, a pattern consistent with prior workplace surveys in Ethiopia [15, 18, 22] and hospital-based studies in Bahrain [40], Qatar [25], Iran [27], India [33], and Turkey [29, 38, 39]. This trend likely reflects their overrepresentation in high-risk manual sectors such as construction and manufacturing (12, 13) Furthermore, sociocultural norms in which men serve as the primary income earners in many Ethiopian households [43]. These findings underscore the socioeconomic implications of productivity loss among this demographic.

Elementary occupation workers had a high injury burden, with notably higher severe injury rates than other occupational groups. In Ethiopia, these workers are often daily laborers or assistants [44] are frequently assigned to high-risk tasks in low-safety environments with limited training or protective equipment [14]. Similar patterns have been reported in Iran [27] and Qatar [25], where manual laborers accounted for the largest share of injuries. These findings highlight the need for targeted safety interventions for high-risk occupations.

Upper extremities were the most frequently affected body region, consistent with workplace surveys in Ethiopia [14, 21] and ED-based studies in India [30], Turkey [37], and Bahrain [40]. This likely reflects the central role of hands and arms in industrial activities, making them vulnerable to injury. Lacerations were the most common injury type, aligning with previous reports [30, 37, 38, 45], and may be explained by frequent exposure to sharp tools, heavy machinery, and cutting equipment in manufacturing [11] and construction settings [45]. These findings underscore the importance of machine guarding, tool safety, and appropriate protective equipment.

Nearly half of the injured patients had low educational attainment. Workers with no formal education or only primary education had a significantly higher prevalence of severe injury compared with those with secondary education or above. This is consistent with prior findings linking lower education to higher injury risk [14], likely due to reduced safety awareness and limited ability to implement preventive measures [46]. Additionally, workers with higher education often occupy supervisory roles with less physical risk. These findings emphasize the need for targeted safety training for less educated workers.

Severe injuries were more prevalent among workers injured outside fixed workplace premises, although the association was modest and marginally significant. This may reflect greater exposure to unfamiliar environments and unfavorable safety climates, which increase the risk of severe injuries in uncontrolled settings [20, 47]. Peer-related and organizational factors were also significantly associated with severe injuries. Similar findings have been reported elsewhere, where co-worker negligence [35] and organizational shortcomings such as poor supervision, inadequate safety protocols, and weak safety culture contribute to severe injuries [11–13, 15–17, 20–22]. Work environments that discourage adherence to safety rules or lack mutual support among workers are known to experience higher injury rates [47]. These findings highlight the critical role of workplace safety culture, compliance, and management accountability in injury prevention.

Mechanisms of injury also influenced severity. Workers who fell or collided with objects had a 77% higher prevalence of severe injury compared with those injured by contact with sharp objects. This aligns with findings from Jordan [41], where falls among construction workers frequently resulted in severe outcomes. Falls from height transfer substantial energy, often causing fractures, head trauma, and internal injuries [42].

Injury severity varied across hospitals, with ALERT and AaBET hospitals reporting the highest proportions of moderate and severe injuries. These facilities function as referral centers and manage more complex trauma cases, likely explaining the observed patterns. In contrast, TBGH hospital received a higher proportion of minor injuries, possibly reflecting differing referral pathways or its role in managing less severe trauma. This variation underscores the need to strengthen healthcare capacity for timely, equitable, and tiered trauma care. Hospitals also serve as critical data sources for occupational injury surveillance, and integrating ED-based data into national monitoring systems could substantially improve prevention efforts.

Overall, this multicenter study provides important evidence on occupational injuries in urban trauma hospitals in Ethiopia. The disproportionate burden among young male workers, those in construction and manufacturing sectors, and individuals with lower educational levels underscores the need for targeted safety interventions, improved risk communication, and stronger enforcement of occupational safety regulations. The strong association of severe injuries with organizational failures, peer-related factors, and fall mechanisms reflect structural weaknesses in safety culture, supervision, and compliance. These insights are valuable for policymakers, employers, and labor authorities in Ethiopia and similar low- and middle-income countries. Future research should expand to rural settings, incorporate long-term outcomes such as disability and return-to-work trajectories, evaluate workplace safety programs, and explore psychosocial hazards and safety culture.

### Study strengths and limitations

This study comprehensively assessed occupational injuries and followed patients from hospital admission through discharge. This prospective approach enabled systematic and timely data collection, minimizing missing information and recall errors. The prospective design and clinical data integration allowed us to capture severe, hospitalized cases often missed by workplace surveys, providing a more complete picture of the trauma burden. Furthermore, the large sample size and reliance on primary data strengthened the validity and reliability of the findings. Data were obtained through structured interviews conducted by trained research assistants across multiple hospitals, ensuring consistency and completeness. To address methodological challenges, assistants received extensive training and used a standardized protocol manual that was developed prior to data collection. Language diversity in Ethiopia posed a potential barrier; this was mitigated by conducting interviews in two widely spoken languages and employing bilingual assistants. The study collected detailed socio-demographic, occupational, accident-related, and injury-specific information, providing a comprehensive understanding of occupational injury patterns and severity.

However, the study was limited to urban public hospitals in Addis Ababa, which may underrepresent occupational injuries in rural areas where work conditions, safety culture, and access to healthcare differ. High-risk populations, treated in private hospitals or those who did not seek care were likely missed, potentially affecting generalizability. Nevertheless, the findings clearly demonstrate the magnitude of occupational injury, the urgent need for safety improvements in the manufacturing and construction sectors, and the utility of hospitals as reliable sources of occupational injury data.

The use of self-reports to describe the accidents may have introduced recall or attribution bias, as participants could adjust narratives to avoid blame or provide socially acceptable explanations, potentially overstating external causes of injuries, e.g. organization or peer-related factors, while underreporting personal or behavioral contributors. Interviewers minimized this risk by employing non-leading questions, inquiring about specific details, and allowing sufficient time for accurate recall.

Finally, injury severity classification relied on clinical judgment, which may introduce some subjectivity and variability in how injuries are categorized across clinicians and facilities. However, the use of the WHO injury severity scale provided a standardized and practical framework for consistent categorization.

## Conclusions

One out of five injury patients registered at EDs had occupational injuries. These injuries disproportionately affected young males with lower educational attainment, particularly those employed in the manufacturing and construction sectors. One-fifth of these injuries were classified as severe, with the mortality rate exceeding what has been reported in comparable studies in other countries. Severe injuries were more likely to occur among workers with low education, from fall-related mechanisms, and in a non-fixed work area. Also, severe injuries are influenced by peers and the organization of the work. These findings highlight the necessity for targeted interventions to enhance workplace safety and health.

## Supplementary Information

Below is the link to the electronic supplementary material.

Supplementary Material 1

Supplementary Material 2

Supplementary Material 3

## Data Availability

The data are available as a minimal dataset from the corresponding author upon reasonable request.

## Abbreviations

AaBET: Addis Ababa Burn, Emergency, and Trauma
ALERT: All Africa Leprosy Rehabilitation and Training Center
APR: Adjusted prevalence ratio
CI: Confidence interval
CPR: Crude prevalence ratio
ED: Emergency departments
ICU: Intensive care unit
ILO: International Labor Organization
IQR: Interquartile range
ISCO: International Standard Classification of Occupations
ISIC: International Standard Industrial Classification
LMIC: Low- and Middle-Income Countries
OSH: Occupational safety and health
PPE: Personal protective equipment
PR: Prevalence ratios
SD: Standard deviation
TBGH: Tirunesh Beijing General Hospital
VIF: Variance inflation factor
WHO: World Health Organization
