# Supplementary material for: Magnitude, characteristics, and factors associated with severe occupational injuries among patients presenting to emergency departments in Addis Ababa, Ethiopia
Source: BMC Emerg Med. 2026 Jun 11;26:222. doi: 10.1186/s12873-026-01643-3 (PMC13488590; doi:10.1186/s12873-026-01643-3)
Supplement: Supplementary file 1 [file 12873_2026_1643_MOESM1_ESM.docx]

**Occupational injuries among emergency department patients in three Ethiopian hospitals: a prospective observational study**

***Part I: Occupational Injury Surveillance tool***

| **No** | **Questions** | **Responses** |
| --- | --- | --- |
| **100** | **General information** |  |
| 101 | Hospital Name*R |  |
| 102 | Name of Interviewer/ ID*R | Text:.______________ |
| 103 | Patient ID (MRN)*R | Number:____________ |
| 104 | Patient COD*R | Text and number: _____________________ |
| 105 | Respondent*R | 01. Injured patient 02. Patient with assistance from the caregiver (Proxy) 03. Caregiver (Proxy) |
| 106 | Date of arrival to hospital (Ethiopian calendar) *R | day/month/year |
| 107 | Time of Arrival (Ethiopian calendar) *R | 01. Morning___hour___minute 02. Afternoon___ hour ___minute 03. Evening___ hour ___minute 04. Night___ hour ___minute |
| 108 | Transferred from another facility*R | 01. Yes 02. No |
| 109 | Dead on arrival*R | 01. Yes 02. No |
| **200** | **Patient Information** |  |
| 201 | Age*R | Number: __________ |
| 202 | Sex*R | 01. Male 02. Female |
| 203 | Educational status*R | 01. Unable to read and write 02. Can read and write but no formal education. 03. Primary school (1-8 grade) 04. Secondary School (9-12 grade) 05. Technical School Certificate/ Diploma 06. Degree and above |
| 204 | Marital Status*R | 01. Single 02. Married 03. Cohabited 04. Separated 05. Divorced 06. Widowed |
| 205 | Number of total persons living in a household, including patient*R | Number: __________ |
| 206 | Residential region*R | 01. Addis Ababa 02. Oromia 03. Amhara 04. Sidama 05. Hadiya, Kembata, Tembaro, Silte, Gurage, Yem 06. Afar 07. Other__________ |
| 207 | Do you live in an urban or rural area?*R | 1. Urban 2. Rural |

| **No** | **Questions** | **Responses** | | |
| --- | --- | --- | --- | --- |
| **300** | **Organization and occupation** |  | | |
| 301 | Did you have more than one job (including a part-time job) during the injury accident? | 01. Yes 02. No |  | |
| 302 | The type of company /organization /sector did you work in during the accident*R  NB. If a worker has more than one job,   - Main job (1-7) - Second job (8-14) - If not construction and Manufacturing skip to Q305 | 01. Agriculture 02. Merchant 03. Construction 04. Manufacturing 05. Civil service 06. Informal sector 07. Other _________ | 08. Agriculture 09. Merchant 10. Construction 11. Manufacturing 12. Civil service 13. Informal sector 14. Other ______ | |
| 303 | If construction, what kind of construction? Please specify. | 01. Building 02. Road 03. other_________ | | |
| 304 | If Manufacturing, what kind of Manufacturing? Please specify. | 01. Woodwork 02. Metalwork 03. Wood and metalwork 04. Textile and Garment 05. Other___________________ | | |
| 305 | The type of Occupation you work *R  NB: If the worker has more than one job,   - Apply 1-11 for Main Job - Apply 12-22 for the Second job | 1. Farming 2. Driver personnel 3. Machine operators 4. Mechanic/assembler 5. Manager or administrator 6. Professionals 7. Technical personnel 8. Salesperson or merchant or shop 9. Personal services, personal care, and protective service worker 10. Elementary, including manual labor 11. Others__________ | | 12. Farming 13. Driver personnel 14. Machine operators 15. Mechanic/assembler 16. Managers or administrator 17. Professionals 18. Technical personnel 19. Salesperson or merchant or shop 20. Personal services, personal care, and protective service workers 21. Elementary, including manual labor 22. Others__________ |
| 306 | Ownership of the organization *R  NB: If the worker has more than one job   - Apply 1-4 for Main Job - Apply 5-8 for the Second job | 1. Government 2. Private/corporate 3. Self/families 4. Other ____________ | | 5. Government 6. Private/Corporate 7. Self/families 8. Other ______ |
| 307 | Employment Status*R  NB: If the worker has more than one job   - Apply 1-6 for Main Job - Apply 7-12 for the Second job | 1. Permanent 2. Temporary or contractual agreement 3. On-call 4. Day laborers 5. Self/families 6. Other ______ | | 7. Permanent 8. Temporary or contractual agreement 9. On-call 10. Day laborers 11. Self/families 12. Other ______ |
| 308 | In which of the jobs you mentioned earlier were you working at the time of the accident? | 01. Main job 02. Second job | | |
| 309 | Working experience on the current job in which the accident occurred *R | Year_________month_________days________ | | |
| **400** | **Injury detail I** |  | | |
| 401 | Day of accident*R | 01. Sunday 02. Monday 03. Tuesday 04. Wednesday 05. Thursday 06. Friday 07. Saturday 08. Unknown | | |
| 402 | Date of accident (Ethiopian calendar) *R | day/month/year_________፟/________፟/__________ | | |
| 403 | Time of accident (Ethiopian time) *R | 01. Morning___hour___minute 02. Afternoon___ hour ___minute 03. Evening___ hour ___minute 04. Night___ hour ___minute | | |
| 404 | Location of accident*R | 01. At usual work area in the establishment (NB. including farm or holding is usual work area for agricultural workers) 02. In the establishment but not usual work area 03. Somewhere else outside the establishment but not usual work area 04. At the usual work area or no fixed work area (eg. street vendors, shoe-shiners, taxi drivers or assistants etc.) 05. Other________________________________________________ | | |
| 405 | How did the injury occur? *R | 01. Contact with sharp/pointed/rough element 02. Trapped, crushed 03. Stuck by something 04. Collided with something 05. Fall at height 06. Fall from the same level 07. Contact with hot object/fire/flame 08. Contact with electric voltage 09. Contact with Chemical 10. Suffered acute overloading of body 11. Drowning, buried 12. Received kick or bite from an Animal 13. Received kick or bite from man 14. Other reason _______________________ | | |
| 406 | Agent of injury*R | 01. Machines 02. Hand tools/ equipment 03. Slip, Trip, and Fall 04. Buildings, structures 05. Materials, objects 06. Conveying, transporting, and packaging equipment excluding vehicles 07. Vehicles 08. Hot object/fire/Flame 09. Electric Voltage 10. Chemical substances 11. Overexertion 12. Animals 13. Humans 14. Other_______________________ | | |
| 407 | Did the task require personal protective Equipment/safety equipment at the time of injury | 01. Yes 02. NO if no go to Q 409 | | |
| 408 | If Yes, Did use of personal protective Equipment/safety equipment at the time of injury?*R | 01. No 02. Glove 03. Helmet 04. Safety shoe 05. Goggles/face shield 06. shield 07. Safety belt 08. Other_______ | | |
| 409 | What do you believe was the primary cause of the accident*? **R  (Choose one that is considered the main cause) *R | 01. Lack of training 02. Lack of work experience 03. Worker fatigue 04. My negligence 05. Other workers' errors/distraction or error in judgment 06. Poor maintenance of equipment 07. Other ________ | | |
| 410 | Was care provided at the scene of injury? *R | 01. Yes 02. No | | |
| 411 | How long did it take you to receive your first medical treatment at any health facility after the accident? *R | Hours : ________Minutes_______ | | |
| 412 | Chronic health conditions that last 1 year or more and require ongoing medical attention *R | 01. No 02. Hypertension 03. Vascular disease 04. Diabetes (type I or type II) 05. Heart attack/disease 06. **Arthritis (arthritis or rheumatoid arthritis)** 07. Bone loss/fracture 08. Degenerative disc disease (back/spinal pain) 09. Neurological diseases 10. Depression/ Anxiety or mood disorders 11. Gastrointestinal disease or digestive tract disease 12. Stroke 13. Asthma 14. Chronic respiratory/lung disease 15. Any allergies 16. Other___________________ | | |

*R: Required
