# Supplementary material for: Magnitude, characteristics, and factors associated with severe occupational injuries among patients presenting to emergency departments in Addis Ababa, Ethiopia
Source: BMC Emerg Med. 2026 Jun 11;26:222. doi: 10.1186/s12873-026-01643-3 (PMC13488590; doi:10.1186/s12873-026-01643-3)
Supplement: Supplementary file 2 [file 12873_2026_1643_MOESM2_ESM.docx]

**በሆስፒታል ውስጥ የሚሰበሰብ የስራ ላይ ጉዳት**

| **ተ.ቁ** | **መጠይቅ** | **ምላሽ** | | |
| --- | --- | --- | --- | --- |
| **100** | ***ጠቅላላ መረጃ*** |  | | |
| 101 | *የሆስፒታሉ ስም *R* |  | | |
| 102 | የመረጃ ሰብሳቢዉ ስም *R | በፊደል:.___________________________ | | |
| 103 | የታካሚዉ የህክምና መለያ ቁጥር (MRN)*R | ቁጥር:____________________________ | | |
| 104 | የታካሚዉ መለያ ቁጥር *R | ፊደል እና ቁጥር:________________________ | | |
| 105 | መረጃዉን የሰጠዉ ግለሰብ*R | 01. በአደጋ ተጎድቶ ለህክና የመጣዉ ታካሚ 02. ታካሚዉ እና ከታካሚዉ ጋር አብሮ ያለ የቅርብ አጋዥ/ተጠሪ 03. ከታካሚዉ ጋር አብሮ ያለ የቅርብ አጋዥ/ተጠሪ | | |
| 106 | ሆስፒታል የመጣበት/ችበት ቀን *R | በኢትዮጵያ አቆጣጥር ቀን/ወር/አመት፡____፟/_____/_______፟ | | |
| 107 | የመጣበት ሰአት (ኢትዮጵያ አቆጣጥር) *R | 01. ጥዋት _____________ሰዓት_______________፟ደቂቃ 02. ከሰአት _____________ሰዓት_______________፟ደቂቃ 03. ምሽት _____________ሰዓት_______________፟ደቂቃ 04. ለሊት_____________ሰዓት_______________፟ደቂቃ | | |
| 108 | ከሌላ ጤ/ተቋም በሪፈራል የመጣ/ች ነዉ/ናት? *R | 01. አዎ 02. አደደለም | | |
| 109 | ወደ ህክምና ተቋሙ ሲደርስ ህይወቱ ያለፈ*R | 01. አዎ 02. አደለም | | |
| **200** | **የታካሚ መረጃ** |  | | |
| 201 | እድሜ*R | ቁጥር:__________________________፟. | | |
| 202 | ጾታ*R | 01. ወንድ 02.ሴት | | |
| 203 | የትምህርት ደረጃ*R | 01. ማንበብና መጻፍ የማይችል/ትችል 02. ማንበብና መጻፍ የሚችል/ትችል ግን በደበኛ ትምህርት ያለተማረ/ች 03. አንደኛ ደረጃ (1-8 ክፍል) 04. 2ኛ ደረጃ (9-12 ክፍል) 05. ቴክኒክና ሙያ ሰረትፊኬት/ዲፕሎማ 06. ዲግሪና ከዚያ በላይ | | |
| 204 | የጋብቻ ሁኔታ*R | 01. ያላገባ/ች 02. ያገባ/ች 03. ህጋዊ ጋብቻ ሳይኖር አብሮ የሚኖሩ 04. ህጋዊ ጋብቻ ያለዉ/ላት ግን አብረዉ የማይኖሩ 05. የፈታ/ች 06. ሚስቱ/ባሏ የሞተችበት/የሞተባት | | |
| 205 | ጠቅላላ የቤተሰብ ብዛት ተጎጅዉን ጨምሮ*R | ቁጥር:__________________________ | | |
| 206 | የሚኖርበት/የምትኖርበት ክልል*R | 01. አዲስ አበባ 02. ኦሮሚያ 03. አማራ 04. ሢዳማ 05. ሀዲያ፣ከምባታ፣ጠምባሮ፣ስልጤ፣ጉራጌ፣የም 06. አፋር 07. ሌላ________________________________________________________ | | |
| 207 | የሚኖሩት ከተማ ነው ውይስ ገጠር? | 01. ከተማ 02. ገጠር | | |
| **300** | **ድርጅት እና ስራ መረጃ** | 01. | | |
| 301 | በአደጋው ወቅት ትርፍ ሰአትን ጨምሮ ከአንድ በላይ የሥራ ቦታ ይሰሩ ነበር? | 01. አዎ 02. አልሰራም | | |
| 302 | ይሰሩ የነበረው የድርጅት ዓይነት *R  ማሳሰቢያ፡ ሰራተኛው ከአንድ በላይ የስራ ቦታ ኧየሰሩ ከሆነ   - ለዋና ስራ 1-7 ይጠቀሙ - ለሁለተኛ ስራ 8-14 ይጠቀሙ - ግንባታ ና የማምረት ካልሆን ውደ ጥያቄ 305 | 01. ግብርና 02. ንገድ 03. ግንባታ 04. ማምረት/ማንፋክቸር 05. ሲቪል ሰርቪስ 06. መደበኛ ያልሆነ ዘርፍ 07. ሌላ______________________ | 08. ግብርና 09. ንገድ 10. ግንባታ 11. ማምረት/ማንፋክቸር 12. ሲቪል ሰርቪስ 13. መደበኛ ያልሆነ ዘርፍ 14. ሌላ_____________________________________ | |
| 303 | ግንባታ ከሆነ የምን ግንባታ? ይጥቀሱ | 01. ህንጻ 02. መንግድ 03.ሌላ____________________________ | | |
| 304 | ማምረት/ማንፋክቸር ከሆነ የምን? ይጥቀሱ | 01. ኧንጨት ስራ 02. ብረት ስራ 03. ኧንጨት ና ብረት ስራ 04. ጨርቃጨርቅ ና ልብስ ስፌት   05,ሌላ________________________________________________ | | |
| 305 | ይሰሩ የነበረው የስራ አይነት*R  ማሳሰቢያ፡ ሰራተኛው ከአንድ በላይ ስራ ካለው   - ለዋና ስራ 1-11 ይጠቀሙ - ለሁለተኛ ስራ 12-22 ይጠቀሙ | 01. ግብርና 02. ሹፌር 03. የማሽን ኦፕሬተር 04. መካኒክ / ጥገና 05. አስተዳዳሪ ወይም የሀላፊ የስራ መደብ 06. ባለሙያ /ፕሮፌሽናል 07. የቴክኒክ ሠራተኞ 08. ሻጭ ወይም ነጋዴ ወይም ሱቅ 09. የግል አገልግሎቶች፣ የግል እንክብካቤ እና የጥበቃ አገልግሎት ሠራተኛ 10. ዝቅተኛ የስራ መደብ የጉልብት ስራ ጨምሮ 11. ሌላ ________________ | | 12. ግብርና 13. ሹፌር 14. የማሽን ኦፕሬተር 15. መካኒክ / ጥገና 16. አስተዳዳሪ ወይም የሀላፊ የስራ መደብ 17. ባለሙያ /ፕሮፌሽናል 18. የቴክኒክ ሠራተኞ 19. ሻጭ ወይም ነጋዴ ወይም ሱቅ 20. የግል አገልግሎቶች፣ የግል እንክብካቤ እና የጥበቃ አገልግሎት ሠራተኛ 21. ዝቅተኛ የስራ መደብ የጉልብት ሠራተኞን ጨምሮ 22. ሌላ_______ |
| 306 | ይሰሩ የነበረበት የድርጅቱ ባለቤትነት *R  ማሳሰቢያ፡ ሰራተኛው ከአንድ በላይ ስራ ካለው   - ለዋና ስራ 1-4 ይጠቀሙ - ለሁለተኛ ስራ 5-8 ይጠቀሙ | 01. የመንግስት 02. የግል/የማህበር ድርጅት 03. የራስ/የተጎጅዉ ቤተሰብ 04. ሌላ_______________________ | | 05. የመንግስት 06. የግል/የማህበር ድርጅት 07. የራስ/የተጎጅዉ ቤተሰብ 08. ሌላ_______________________ |
| 307 | ይሰሩ የነበረበት የቅጥር ሁኔታ *R  ማሳሰቢያ፡ ሰራተኛው ከአንድ በላይ ስራ ካለው   - ለዋና ስራ 1-6 ይጠቀሙ - ለሁለተኛ ስራ 7-12 ይጠቀሙ | 01. ቋሚ 02. በኮንትራት/ጊዜያዊ ስምምነት 03. በጥሪ 04. የቀን ሰራተኛነት 05. የራስ /የተጎጅዉ ቤተሰብ 06. ሌላ_______________________ | | 07. ቋሚ 08. በኮንትራት/ጊዜያዊ ስምምነት 09. በጥሪ 10. የቀን ሰራተኛነት 11. የራስ /የተጎጅዉ ቤተሰብ 12. ሌላ_______________________ |
| 308 | ቀደም ሲል ከጠቀስካቸው/ሽው ሥራዎች ውስጥ አደጋው የደረሰው በየትኛው ሥራ ነበር? *R | 01. ዋና ሥራ 02. ሁለተኛ ሥራ | | |
| 309 | አደጋ ያደረሰብዎትን ስራ ለምን ያህል ጊዜ ሰሩ? *R | አመት___________ ወር___________ቀን____________ | | |
| **400** | **አደጋዉ** **ዝርዝር I** |  | | |
| 401 | የአደጋ ቀን *R | 01. እሁድ 02. ሰኞ 03. ማክሰኞ 04. እሮብ 05. ሀሙስ 06. አርብ 07. ቅዳሜ 08. አላውቅም / አላስታዉስም | | |
| 402 | አደጋዉ የደረሰበት ቀን/ወር/አመት *R | በኢትዮጵያ አቆጣጥር ፡____፟/_____/_______፟ | | |
| 403 | አደጋዉ የደረሰበት ሰአት (በኢትዮጵያ አቆጣጥር) *R | 01. ጥዋት _____________ሰዓት_______________፟ደቂቃ 02. ከሰአት _____________ሰዓት_______________፟ደቂቃ 03. ምሽት _____________ሰዓት_______________፟ደቂቃ 04. ለሊት_____________ሰዓት_______________፟ደቂቃ | | |
| 404 | የአደጋው ቦታ *R | 01. በድርጅቱ ውስጥ በተለመደው ቦታ ግብርናን ጨምሮ የሥራ ቦታ 02. በድርጅቱ ውስጥ በተለመደው ሰራ ቦታ ያልሆነ 03. ከተቋሙ ውጭ የሆነ ሌላ ቦታ በተለመደው የሥራ ቦታ ያልሆነ 04. ከድርጅት/ከተቋት ውጭ ሌላ ቦታ ሆኖ በተለመደው የሥራ ቦታ፣ ውስን የስራ ቦታ ያልሆነ (ምሳ. ምኪና ሹፌር/ኧረዳት ፣የመነግድ ላይ ስራ፣ጫማ መጥረግ፤የመንግድ ዳር ንግድ....) 05. ሌላ____________________________ | | |
| 405 | አድጋው እንዴት ተከሰተ?*R | 01. በሹል/በስለት/በዱልዱም ነገር መወጋት/መቆረጥ 02. መጣብቅ/መያዝ 03. በሆነ ነገር መመታት (ምሳሌ፡ በሚውረወር፣በሚፈናጠር፣ከከፍታ ላይ በወደቀ፣በተንጠለጠለ ነገር ና በተንከባለለ ነገር መመታት) 04. ከሆነ ነገር ጋር መጋጨት 05. ከከፍታ ላይ መውድቅ 06. ከተመሳሳይ ወለል/ሜዳ ላይ መውደቅ 07. ትኩስ ነገር / እሳት / ነበልባል ጋር የተገናኘ 08. ከኤሌክትሪክ ቮልቴጅ ጋር የተገናኘ 09. ከአደገኛ ንጥረ ነገር /ኪሚካል ጋር የተገናኘ 10. በሰውነት ላይ ከፍተኛ ጭነት ማሳረፍ 11. መስጠም ፣ መቀበር 12. በእንስሳ መመታት፣ መነከስ 13. በሰዉ መመታት፣ መነከስ 14. ሌላ___________________________ | | |
| 406 | የአደጋዉ ምክንያት*R | 01. ማሽን 02. የእጅ መሳሪያ 03. መንሸራተት፣ መደናቀፍ እና መውደቅ 04. የህንጻ አካል/የግንባታ አካል 05. በእቃ/በማንኛዉም ነገር መመታት 06. የሚያጓጓዝ መሳሪያዎች / የሚያጓጓዝ ማሽን ከመኪና ዉጭ 07. መኪና 08. እሳት/ትኩስ ነገር 09. ኤሌክትሪክ 10. የኬሚካል ንጥረ ነገሮች 11. ሰውነትን ከአቅም በላይ ለሆነ ከብድት ማጋለጥ 12. እንስሳ 13. ሰዉ 14. ሌላ__________________________________ | | |
| 407 | በአደጋውን ሊከላከል የሚችል የአደጋ ምከላከያ ልብስ (personal protective equipment)/ቀበቶ ያስፈልገዉ ነብር? | 01. አዎ ስራዉ የአደጋ ምከላከያ ያስፈልገዋል 02. ስራዉ የአደጋ ምከላከያ አይፍልግም ---------------- አይፍልግም ክሆነ ወደ ተ.ቁ 409 ይሂዱ› | | |
| 408 | አዎ ከሆነ በአደጋው ወቅት የአደጋ ምከላከያ ተጠቅመዋል? *R | 01. አልተጠቀምኩም 02. የኧጅ ግዋንት 03. የራስ ቅል መከላከያ ኮፍያ 04. ሰፍቲ ጫማ 05. የአይን/ፊት መከላክያ 06. የደህንነት ቀበቶ 07. ሌላ__________________________________ | | |
| 409 | የአደጋዉ ዋነኛ ምክንያት ምንድነው ብለው ያስባሉ?*R  (እንደ ዋና መንስኤ የሚቆጠረውን አንዱን ይምረጡ) | 01. የሰራ ላይ ድህንነት ስልጠና ያለማግኘት 02. የስራ ልምድ ማንስ 03. በጣም ደክሞኝ ስለነበር 04. የእኔ ቸልተኝነት 05. የሌላ ሰዉ ስተት/ምክንያት 06. ባግባቡ ጥገና ባለመኖሩ/ባለመደረጉ 07. ሌላ__________________________________ | | |
| 410 | ጉዳት በደረሰበት ቦታ ላይ የመጀመሪያ ህክምና እርዳታ አግኝተዉ ነበር? *R | 01. አዎ 02. አደለም | | |
| 411 | አደጋ ከደረሰብዎት በኋላ በማንኛውም የጤና ተቋም የመጀመሪያዉን ህክምና በስንት ሰአት ጊዜ አገኙ *R | ሰአት: ________ደቂቃ_______________ | | |
| 412 | በሀኪም የተርጋገጠ አንድ አመትና ከዛ በላይ በሀኪም ክትትል የሚያደርጉት ሥር የሰደደ ሕመም/በሽታ አለብዎት *R | 01. የለም 02. የደም ግፊት 03. የደም ቧንቧ በሽታ 04. የስኳር በሽታ (ዓይነት I ወይም II ዓይነት) 05. የልብ ህመም/በሽታ 06. የቁርጥማት በሽታ (አርትራይተስ ወይም የሩማቶይድ እና የአርትሮሲስ) 07. የአጥንት መሳሳት/መበሳሳት 08. የዲስክ፤ የጀርባና መገጣጠሚያ ህመም 09. የነርቭ በሽታዎች 10. ድብርት/መደበትስትሮክ 11. የጨጓራና የአንጀት በሽታ ወይም የምግብ ማስተላለፊያዎች ህመም 12. ስትሮክ/የጭንቅላት ውስጥ ደም መፍሰስ 13. አስም 14. ሥር የሰደደ የመተንፈሻ አካል/የሳንባ በሽታ 15. ማንኛውም አለርጂ(ይግለጹ_________________) 16. ሌላ_________________________________________ | | |

R: አስፈላጊ/የማይዘለል
