# Supplementary material for: Magnitude, characteristics, and factors associated with severe occupational injuries among patients presenting to emergency departments in Addis Ababa, Ethiopia
Source: BMC Emerg Med. 2026 Jun 11;26:222. doi: 10.1186/s12873-026-01643-3 (PMC13488590; doi:10.1186/s12873-026-01643-3)
Supplement: Supplementary file 3 [file 12873_2026_1643_MOESM3_ESM.docx]

**Occupational injuries among emergency department patients in three Ethiopian hospitals: a prospective observational study**

**Afan Oromo version of Occupational Injury Surveillance Tool**

**Hospitaala keessatti raga midhama hojiin wal-qabate iaalchisee sassaabamu.**

| **Lak.** | **Gaaffii** | **Deebii** |
| --- | --- | --- |
| ***100*** | ***Odeeffannoo waliigallaa*** |  |
| 101 | Maqaa hospitaalaa *R |  |
| 102 | Maqaa gaafataa/adda baaftuu *R | Barreeffamaan ……………………….. |
| 103 | Lakkoofsa kaardii dhukubsataa*R | Lakkoofsaan ……………………………. |
| 104 | Koodii dhukkubsataa*R | Barreeffamaafi lakkoofsa…………………… |
| 105 | Gaafatamaa*R | 01. Dhukkubsataa miidhaan irra gahe 02. Dhukkubsataaf gargaaraa isa wajjin(bakka bu’aa) 03. Gargaaraa dhukkubsataa (bakka bu’aa) |
| 106 | Guyyaa itti hospitaala gale/te (Kalaandara itiyoopiyaa)*R | Guyyaa/ji’a/wagga………………………… |
| 107 | Yeroo itti hospitaala dhufe (Akka Itiyoopiaatti) *R | 01. Ganama___sa'aatii___daqiiqaa  02. Waaree booda___ sa’aatii ___daqiiqaa  03. Galgala___ sa'aatii ___daqiiqaa  04. Halkan___ sa’aatii ___daqiiqaa |
| 108 | Mana yaalaa biro irraa ergamee/riferiin kan dhufeedhaa? *R | 01. Eeyyee 02. Lakkii |
| 109 | Osoo gara hospitaalaa dhufuu lubbuun kan darbe? *R | 01. Eeyyee 02. Lakkii |
| **200** | **Ragaa nama yaalamuu** |  |
| 201 | Umurii *R | Lakkoofsa…………………………….. |
| 202 | Saala *R | 01. Dhiira 02. Dhalaa |
| 203 | Sadarkaa barnoota*R | 01. Barreessuuf dubbisuu kan hin dandeenye 02. Barreessuuf dubbisuu danda’ee garuu barnoota idilee hin qabu 03. Sadarkaa jalqabaa (1-8) 04. Sadarkaa lammataa (9-12) 05. Sertifikeetii mana barumsaa Teekinikaa /diploomaa 06. Digirii jalqabaaf isaa ol |
| 204 | Haala gaa’elaa*R | 01. Kan hin fudhiin/herumiin 02. Gaa’ela kan qabu/du 03. Walumaan jireenya qofa/ga’ela idlee kan hin qabne 04. Addaan kan bahe/te, ga’ela idlee kan qaban 05. Kan hike/te 06. Kan jalaa du’e/te |
| 205 | Lakkoofsa waliigala namoota mana tokko keessa jiraatan, dhukkubsataa dabalatee*R | Lakkoofsa__________ |
| 206 | Naannoo bakka jireenyaa*R | 01. Finfinnee 02. Oromiyaa 03. Amaara 04. Sidaamaa 05. Haadiyaa, kambaataa, Xambaaroo, Silxee, Gurage, Yem 06. Afar 07. Kan biro___________________ |
| 207 | Iddon jirenya keessan magaala moo baadiyaa? | 01. Magaalaa 02. Baadiya |

| **Lak.** | **Gaaffiilee** | **Deebii** | | |
| --- | --- | --- | --- | --- |
| **300** | **Waajjiraafi odeeffannoo haala hojii** |  | | |
| 301 | Yeroo midhaan isin irra ga’e hojii tokko ol (hojii dhaabbataa hin ta’iin dabalatee) hojjettu turtanii? | 01. Eeyye 02. Lakkii | | |
| 302 | Gosa dhaabbata /gurmaa'ina /sector yeroo balaa sanaa hojjettee*R  **Hubachiisa (Hub).**  • Hojjetaan tokko hojii tokkoo ol yoo qabaate 1 hojii ijoo (1-7) yoo ta’u 2 ammoo hojii lammaffaa (8-14) .  • Yoo ijaarsaa fi Omisha hin taane gara Q305^tti^ darbi | 01. Qonnaa 02. Daldala 03. Ijaarsa 04. Oomishaa/manufaakicharii 05. Siivil seervisii 06. Dame al-seerummaa 07. Kan biro _______ | 08. Qonnaa 09. Daldala 10. Ijaarsa 11. Oomishaa/manufaakicharii 12. Siivil seervisii 13. Damee al-seerummaa 14. Kan biraa   ___ | |
| 303 | Yoo Ijaarsa ta’e, ijaarsa akkamii akka ta’eibsi | 01. Ijaarsa Gamoo 02. Karaa/daandii 03. Kan biraa ____________ | | |
| 304 | Yoo Oomisha/manufaakicharii ta’e, Oomisha akkamii akka ta’eibsi | 01. Hojii mukaa 02. Hojii sibilaa 03. Hojii mukaa fi sibilaa 04. Warshaa ufataa fi hodhaa 05. Kan biraa ibsii ______________ | | |
| 305 | Gosa Hojii yeroo balaa sanaa hojjettu *R  Hub: Hojjetaan hojii tokkoo ol yoo qabaate,   - 1-11 hojii ijoof guuti - 12-22 hojii Lammaffaa guuti | 01. Qonna/omisha 02. Koonkolaachisaa 03. Opreetara maashinaa 04. Hoji makaanikki/suphaa 05. Hoji bulchinsa/itti gafatamaa 06. Ogessa/profeshinaala 07. Hoji teknikaa 08. Hoji gurgurtaa/daldala 09. Tajaajila dhuunfaa, kunuunsa dhuunfaa, fi hojjettoota tajaajila eegumsaa 10. Hoji gad-aanaa, hojii humnaa dabaltee 11. Kan biraa (ibsi) _______________ | | 12. Qonna/omisha 13. Koonkolaachisaa 14. Opreetara maashinaa 15. Hoji makaanikki/suphaa 16. Hoji bulchinsa/itti gafatamaa 17. Ogessa/profeshinaala 18. Hoji teknikaa 19. Hoji gurgurtaa/daldala 20. Tajaajila dhuunfaa, kunuunsa dhuunfaa, fi hojjettoota tajaajila eegumsaa 21. Hoji gad-aanaa, hojii humnaa dabaltee 22. Kan biraa (ibsi) |
| 306 | Abbummaa dhaabbatichaa *R  Hub: Hojjetaan hojii tokkoo ol yoo qabaate   - 1-4 hojii ijoof guuti - 5-8 hojii lammaffaa guuti | 01. Kan mootummaa 02. Kan dhuunfaa/garee 03. Kan dhunfaa/maatii 04. Kan biroo ibsi ______________ | | 05. Kan mootummaa 06. Kan dhuunfaa/garee 07. Kan dhunfaa/maatii 08. Kan biroo ibsi ______________ |
| 307 | Haala qacarrii*R  Hub: Hojjetaan hojii tokkoo ol yoo qabaate   - 1-6 hojii ijoof guuti - 7-12 hojii lammaffaaf guuti | 01. Dhaabbataan 02. Kontoraata/yeroodhaaf 03. Hojjetaa waamichaan hojetu 04. Hojjata guyyaa guyyan hojjatu 05. Kan dhuunfaa/kan maatii yoo ta’e hin laallatu 06. Kan biraa | | 07. Dhaabbataan 08. Kontoraata/yeroodhaaf 09. Hojjetaa waamichaan hojetu 10. Hojjata guyyaa guyyan hojjatu 11. Kan dhuunfaa/kan maatii yoo ta’e hin laallat 12. Kan biraa |
| 308 | Hojiiwwan armaan dura kaaftan keessaa yeroo balaan kun mudate isa kam keessatti hojjechaa turtan? | 01. Hojii ijoo 02. Hojii lammaffaa | | |
| 309 | Hojii amma balaan kun itti mudate irratti muuxannoo hojii qabaachuu | Waggaa ___________ ji’a _________ | | |
| Lak. | Gaaffii | Deebii | | |
| **400** | **Gosa miidhamichaa** |  | | |
| 401 | Guyyaa balaan itti dhaqqabe*R | 01. Dilbata  02. Wiixata  03. Kibxata  04. Roobii  05. Kamisa  06. Jimaata  07. Sambata   01. 08. Hin yaadadhu | | |
| 402 | Guyyaa itti balaan dhaqqabe (kaalaandarii Itiyoophiyaa) *R | ________/_________/____________ | | |
| 403 | Sa’atii itti balaan dhaqqabe (sa'aatii Itoophiyaa) *R | 01. Ganama___sa'aatii___daqiiqaa  02. Waaree booda___ sa’aatii ___daqiiqaa  03. Galgala___ sa'aatii ___daqiiqaa  04. Halkan___ sa’aatii ___daqiiqaa | | |
| 404 | Bakka balaan itti dhaqqabe*R | 01. Bakka hojii barame dhaabbaticha keessatti (NB. qonnaa ykn qabachuu dabalatee bakka hojii barame hojjettoota qonnaati) . 02. Dhaabbata keessatti garuu naannoo hojii barame miti 03. Bakka biraa dhaabbatichaan alatti garuu bakka hojii barame miti 04. Bakka hojii barame ykn bakka hojii dhaabbataa hin jirretti (fkn. daldaltoota daandii irratti, namoota kophee caccabsan, konkolaachiftoota taaksii ykn gargaartota fi kkf) . 05. Kan biroo___ . | | |
| 405 | Balaan akkamitti umamee? *R | 01. Waan qara qabuun waranamu/muramuu 02. Waan wayitiin Qabamuu/maxxanuu 03. Meshaa wayitiin rukutamu 04. Waan wayii waliittii bu’u 05. Waan olkaa’a irraa kufuu 06. Badhee irra kufuu 07. Waan ho’a kan akka abidaa, qaanqee, 08. Volteejii elektriitiin wal qabatee 09. Keemikaala balaa gesissun 10. Wanta/meeshaa guddaa qama irratti bu’u 11. Uwifamuu/awaalamuu 12. Bineensa/beeladaan waraanamu, cininamuu 13. Namaan rukutamuu, cininamu 14. Kan biraa | | |
| 406 | Sababa Balaan gahuu danda’e*R | 01. Maashinii 02. Meeshaa harkaan 03. Mucucaacuu fi kufuu 04. gamoo walii walitti bu’u 05. Meeshalee ykn Wanta ta’en rukutamuu 06. Meeshaa geekjiba/maashinii socho’u garuu osookoonkolaata hin dabalatiin 07. Konkolaataa 08. Waan ho’aa/ibidda/labooba ibiddaa waliin kan wal qabate 09. Electrikii waliin wal qabate 10. Keemikaalota balaafamoo ta’an waliin kan wal qabate 11. Ulfaatina hin mallee kaasuun 12. Beeylad/binensa 13. Nama 14. Kan biro ______________ | | |
| 407 | Yeroo miidhaan isin irra gahe hojiin meeshaalee nageenyaa (personal protective Equipment) barbaachisaa turee? | 01. Eeyyee  02. LAKK yoo lakki ta’e gara Q 409 deemaa | | |
| 408 | Yoo eeyyee ta’e, yeroo balaan gahee sana meshaa ittisa balaa fayadamtanii/godhatanii ture? *R | 01. Lakkii 02. Haguuggi harkaa 03. Meeshaa mataa irratti kaawatamuu 04. Kophee balaa ittisu/safety shoes 05. Meeshaa balaa ijaa/fulaa ittisu 06. Hidhata/qabattoo konkoolataa 07. Kan biraa _____________________ | | |
| 409 | Sababiib balaan kun itti umamuu danda’e inni guddan maalii jettanii amantu? *R  (Kana sababa guddaa ta'ee fudhatame filadhu) | 01. Hanqina leenjii 02. Muuxannoo hojii dhabuu 03. Dadhabbii hojjetaa 04. Dagannoo koo 05. Dogoggora hojjettoota biroo 06. Suphaan meeshaalee gaarii hin taane 07. Kan biraa ___________________ | | |
| 410 | Bakka balaan gahe sanatti tajaajila fayyaa duraa argattaniittuu?*R | 01. Eeyyee 02. Lakkii | | |
| 411 | Dhaabbata fayyaa kam irraas haa ta’u balaan erga gahee booda sa’a meeqa keessatti tajaajila fayyaa duraa argattan?*R | Sa’atii _____________ Daqiiqaa__________________ | | |
| 412 | Ogessa fayaattin kan mirkana’e dhukkuba wagga tokko ol kan ta’ee fi hordoffi itti fufiinsaa barbaachisu qabdu?*R | 01. Lakkii 02. Dhibbaa dhigaa 03. Dhibee ujummoo dhigaa 04. Dhibee sukaaraa 05. Dhibee onne 06. Dhibe qaama caccabsuu (arthritis/rheumatoid arthritis ) 07. Dhibee haphina lafe/caba lafee 08. Dhukkuba diskiin manca’u (dhukkubbii dugdaa/lafee dugdaa) . 09. Dhibee Narvii 10. Cinqamuu 11. Dhukkuba garaachaa ykn dhukkuba ujummoo bullaa'insa nyaataa 12. Dhibee dhigaa hammaataa /stroke/ 13. Asmaa 14. Dhibbee ujummo qileensaa kan akka sombaa 15. Alarjii kamiyyu (ibsii ______________) 16. Kan biraa _____________________ | | |

R jechuun baay’ee barbaachisaa/kan irran in darbamne.
